# Teachers' perspectives on the implementation of the new national curriculum - Dataset from Vietnam

**DOI:** 10.1016/j.dib.2023.109451

**Published:** 2023-07-27

**Authors:** Hue Thi Thu Dang, Dien Thi Bui, Quoc Anh Vuong, Huong Giang Thi Phan, Chi Thi Nguyen, Bich Dao Thi Pham

**Affiliations:** The Vietnam National Institute of Educational Sciences, 101 Tran Hung Dao Street, Hoan Kiem District, Hanoi 100000, Vietnam

**Keywords:** Teachers’ challenges, Teachers’ perceptions, Teacher development, New curriculum, Curriculum implementation, Vietnam education

## Abstract

This dataset provides insightful data on the reality of the new national curriculum implementation from primary and secondary school teachers’ perspectives. The dataset addresses four main aspects of the new curriculum implementation from teachers’ perspectives: (a) teachers' capacity in implementing the new curriculum, (b) teaching conditions in implementing the new curriculum, (c) challenges for teachers in implementing the new curriculum, and (d) teachers' desires to implement the new national curriculum successfully. The survey tools used to collect the data were developed based on a literature review of implementing a new curriculum from teachers’ assessment. The survey was conducted online via Google Forms from November to December 2022, with 29,026 primary and secondary school teachers in five provinces of Vietnam participating. The dataset is expected to benefit policymakers and educational administrators by providing information on key areas where teachers require support and training to improve the implementation of the new curriculum. Additionally, the dataset contributes to developing evidence-based policies and interventions to support teachers in implementing the new curriculum. Educational managers and researchers can benefit from this dataset by gaining insights and preparing adequately for implementing new curriculum or developing plans to improve curriculum development. Overall, this dataset provides valuable information for improving the effectiveness of implementing the new national curriculum.

**Specifications Table**

**Table:** 

Subject	Social sciences
Specific subject area	Education Teacher development Curriculum implementation
Type of data	Tables Figure Excel file
How the data were acquired	The data was collected using an online survey created with Google Forms. The survey link was distributed to primary and secondary school teachers through the educational management system of the five Departments of Education and Training. The responses collected from the teachers were then imported into an Excel spreadsheet for data analysis. Statistical analysis was conducted using SPSS Version 26 to identify trends and patterns in the data.
Data format	Raw Analysed
Description of data collection	The data was collected using the cluster sampling method. Participating teachers were selected from five provinces, including Hung Yen, Lao Cai, Nghe An, Kien Giang, and Ho Chi Minh City. The targeted respondents for the survey were primary school teachers in grades 1, 2, and 3, as well as lower secondary school teachers in grades 6 and 7. A total of 29,026 valid responses were received, with 22,602 female and 6424 male respondents.
Data source location	Institution: The Vietnam National Institute of Educational Sciences City/Town/Region: Hung Yen, Lao Cai, Nghe An, Kien Giang and Ho Chi Minh City Country: Vietnam Latitude and longitude (and GPS coordinates, if possible) for collected samples/data: Hung Yen: 20.8526° N, 106.0170° E Lao Cai: 22.4809° N, 103.9755° E Nghe An: 19.2342° N, 104.9200° E Kien Giang: 9.8250° N, 105.1259° E Ho Chi Minh City: 10.8231° N, 106.6297° E
Data accessibility	Repository name: Mendeley Data Data identification number: 10.17632/rffkw6w8cn.1 Direct URL to data: https://data.mendeley.com/datasets/rffkw6w8cn

## Value of the Data

• The dataset is expected to make methodological and practical contributions to the study of the new curriculum implementation from teachers’ perspectives.
• The dataset provides a large-scale database of primary and secondary school teachers’ evaluations regarding the implementation of the new national curriculum in Vietnam.
• The dataset's findings can be used to develop evidence-based policies and interventions to support teachers in implementing the new curriculum effectively and to enhance the overall quality of education in Vietnam.
• The data collection method uses a survey created based on the theory of educational implementation, as well as a literature review, providing a reliable and valid tool and research process for collecting data.
• The dataset adds empirical evidence to the reality of new curriculum implementation which can benefit future research. Vietnamese and international researchers and educators can use the data to design new curriculum, propose strategies to limit challenges and enhance the effectiveness of new curriculum implementation.

## 1 Data Description

The dataset, which is available on Mendeley data [4], provides valuable insights into the reality of implementation of the new curriculum in Vietnam from teachers’ viewpoints. It includes a comprehensive questionnaire with 64 items, along with a raw data file containing 29,026 responses The raw data consists of two sheets: Sheet 1 contains the codebook, providing the main content of each question and its corresponding coded responses. Sheet 2 contains the raw data with teachers' answers, encoded according to the codebook. The questionnaire is structured into five groups, consisting of (a) demographic information of participating teachers, (b) teachers' capacity in implementing the new curriculum, (c) teaching conditions in implementing the new curriculum, (d) challenges faced by teachers in implementing new curriculum, and (e) teachers' desires to implement the new curriculum successfully. The demographic items are in the form of selected responses, while the remaining items are in the form of 5-point Likert statements.

The first section of collected data pertained to the gender, teaching grade, and place of residence of the participating teachers, as shown in Table 1. Additionally, this section provided information on the age and teaching experience, as well as the teaching qualifications of the respondents. It also indicated whether teachers had received training related to educational aspects such as curriculum, teaching methods, or classroom management. These demographic details can be examined for any correlations with other questionnaire items. The first section includes 15 items, ranging from Question 1 to Question 8 in the codebook.

**Table 1:** Distribution of participating teachers by gender, teaching grade, and geographical location.

	Gender*		Grade					School Location		
Total 29,026	Female	Male	Grade 1	Grade 2	Grade 3	Grade 6	Grade 7	Urban area	Rural area	Mountainous area
	22,602	6424	8561	5242	4718	6386	4119	11,573	12,532	4921

⁎ The gender disparity between female and male teachers in the surveyed population reflects the natural imbalance in the proportion of female and male teachers in Vietnam, as the data was collected using a cluster sampling method. According to the data from the General Statistics Office of Vietnam, in general education, female teachers make up nearly 70% of the total number of teachers, while the remaining 30% are male teachers.

The second group of information, consisting of 12 items, focused on teachers' capacity to implement the new curriculum. This group assessed their understanding of subject objectives, teaching methods, and assessment, as well as their confidence in conducting educational activities. Data on these aspects are presented in Questions 9.1 to 9.4 and Questions 10.1 to 10.8 in the codebook.

In the third section of the questionnaire, consisting of 7 items, teachers were asked to indicate the extent of their satisfaction with the teaching conditions for implementing the new curriculum. This included aspects such as their working hours, salary, and allowances (Questions 11.1 to 11.3). Additionally, they were asked to evaluate the adequacy of school facilities, such as the quality and quantity of classrooms, function rooms, teaching equipment, and learning resources (Questions 12.1 to 12.4).

The fourth group of information focused on the challenges faced by teachers in implementing the new curriculum. It encompassed designing lesson plans, teaching methods, and assessing student learning. Additionally, it explored the factors contributing to difficulties and challenges in adopting the new national curriculum. The data collected in these areas are presented in the raw data file with 23 items, ranging from Questions 13.1 to 13.9 and Questions 14.1 to 14.14 in the dataset. Table 2 illustrates the differences in teachers' challenges in implementing the new curriculum across different regions.

**Table 2:** Differences in teachers’ challenges in implementing new curriculum by region (including Test of Homogeneity of Variances, Welch and ANOVA).

Test of Homogeneity of Variances						
	Levene Statistic	df1	df2	Sig.		
Lack of timely guidelines	1.055	2	29,023	.348		
Teacher unawareness of the new curriculum	1.376	2	29,023	.252		
Teachers’ lack of readiness to implement the new curriculum	.143	2	29,023	.867		
Unsuitable implementation timeline due to COVID-19 Pandemic	.497	2	29,023	.608		
Teachers have not been fully trained.	.087	2	29,023	.917		
Ineffective training courses for Teachers	.942	2	29,023	.390		
Senior teachers struggling to adapt to the new curriculum	.381	2	29,023	.684		
Junior teachers lacking sufficient experience for the new curriculum	.231	2	29,023	.794		
Inadequate learning facilities for students	.288	2	29,023	.750		
large class size	.108	2	29,023	.898		
Inadequate teaching equipment	5.323	2	29,023	.005		
Heavy workload for teachers	1.557	2	29,023	.211		
Lack of support from school administrators	5.400	2	29,023	.005		
Inadequate professional development activities at schools and school clusters	5.144	2	29,023	.006		

ANOVA						

	Sum of Squares	df	Mean Square	F	Sig.	

Lack of timely guidelines	Between Groups	14.617	2	7.308	10.100	.000
	Within Groups	21,000.500	29,023	.724		
	Total	21,015.116	29,025			
Teacher unawareness of the new curriculum	Between Groups	9.353	2	4.677	5.862	.003
	Within Groups	23,152.802	29,023	.798		
	Total	23,162.155	29,025			
Teachers’ lack of readiness to implement the new curriculum	Between Groups	8.474	2	4.237	4.833	.008
	Within Groups	25,445.283	29,023	.877		
	Total	25,453.757	29,025			
Unsuitable implementation timeline due to COVID-19 Pandemic	Between Groups	4.711	2	2.355	3.342	.035
	Within Groups	20,453.786	29,023	.705		
	Total	20,458.497	29,025			
Teachers have not been fully trained.	Between Groups	7.424	2	3.712	4.118	.016
	Within Groups	26,161.238	29,023	.901		
	Total	26,168.662	29,025			
Ineffective training courses for Teachers	Between Groups	4.833	2	2.417	2.955	.052
	Within Groups	23,736.298	29,023	.818		
	Total	23,741.131	29,025			
Senior teachers struggling to adapt to the new curriculum	Between Groups	15.587	2	7.793	9.097	.000
	Within Groups	24,864.083	29,023	.857		
	Total	24,879.669	29,025			
Junior teachers lacking sufficient experience for the new curriculum	Between Groups	28.551	2	14.276	15.831	.000
	Within Groups	26,171.018	29,023	.902		
	Total	26,199.569	29,025			
Inadequate learning facilities for students	Between Groups	6.670	2	3.335	4.198	.015
	Within Groups	23,053.378	29,023	.794		
	Total	23,060.048	29,025			
large class size	Between Groups	124.059	2	62.030	74.550	.000
	Within Groups	24,148.630	29,023	.832		
	Total	24,272.689	29,025			
Inadequate teaching equipment	Between Groups	2.875	2	1.438	1.829	.161
	Within Groups	22,817.173	29,023	.786		
	Total	22,820.048	29,025			
Heavy workload for teachers	Between Groups	99.003	2	49.502	60.968	.000
	Within Groups	23,564.748	29,023	.812		
	Total	23,663.751	29,025			
Lack of support from school administrators	Between Groups	4.176	2	2.088	2.314	.099
	Within Groups	26,189.716	29,023	.902		
	Total	26,193.892	29,025			
Inadequate professional development activities at schools and school clusters	Between Groups	1.164	2	.582	.640	.528
	Within Groups	26,420.635	29,023	.910		
	Total	26,421.799	29,025			
Robust Tests of Equality of Means						

	Statistic^a^	df1	df2	Sig.		

Lack of timely guidelines	Welch	10.150	2	13,446.174	.000	
Teacher unawareness of the new curriculum	Welch	5.834	2	13,617.593	.003	
Teachers’ lack of readiness to implement the new curriculum	Welch	4.826	2	13,575.152	.008	
Unsuitable implementation timeline due to COVID-19 Pandemic	Welch	3.329	2	13,492.840	.036	
Teachers have not been fully trained.	Welch	4.112	2	13,541.254	.016	
Ineffective training courses for Teachers	Welch	2.942	2	13,560.899	.053	
Senior teachers struggling to adapt to the new curriculum	Welch	9.096	2	13,574.478	.000	
Junior teachers lacking sufficient experience for the new curriculum	Welch	15.759	2	13,598.715	.000	
Inadequate learning facilities for students	Welch	4.190	2	13,571.174	.015	
large class size	Welch	73.478	2	13,466.459	.000	
Inadequate teaching equipment	Welch	1.806	2	13,578.635	.164	
Heavy workload for teachers	Welch	60.572	2	13,475.382	.000	
Lack of supports from school administrators	Welch	2.295	2	13,550.774	.101	
Inadequate professional development activities at schools and school clusters	Welch	.651	2	13,602.497	.522	

a Asymptotically F distributed.

The final group of information consisted of 7 items designed to assess teachers' desires to effectively implement the new national curriculum. This data is shown in the raw data file from Question 15.1 to Question 15.7.

## 2 Experimental Design, Materials and Methods

The implementation of a new national curriculum is a major educational transformation that demands significant efforts from all those involved [2,6]. Teachers, as key players, may encounter many challenges in the process [1,5]. Understanding these challenges is essential for improving the effectiveness of educational reforms and ensuring its successful implementation [12]. The main data collection tool was thoughtfully and extensively designed based on several relevant theories and a review of the relevant literature on teachers' assessments of implementing the new curriculum. Firstly, the theory of curriculum implementation suggests that the successful implementation of a new curriculum depends on factors such as teacher capacity, resource availability, and school support [11]. Secondly, the theory of teacher professional development emphasizes the importance of teacher knowledge and skills in successful curriculum implementation [7,10]. Thirdly, the study draws from social cognitive theory, which posits that self-efficacy plays a significant role in the success of individuals in various domains, including teaching [3]. Finally, the study adopts the concept of school effectiveness, which highlights the importance of school resources and conditions in achieving educational goals [8,9].

In terms of sampling, the study targeted teachers in grades 1, 2, 3, 6, and 7 who have been implementing the new curriculum since the 2020–2021 school year. To ensure the currency and validity of the data collection tool, a literature review was conducted, and experts were consulted to inform the questionnaire design. The questionnaire was then adapted into an online survey format with a combination of mandatory and optional questions to be administered on Google Forms. Before being distributed to the Departments of Education and Training for approval to be administered on a large scale, the questionnaire was piloted on 68 teachers and revised for wording and item number to improve its quality. The questionnaire demonstrated high internal consistency, with a Cronbach's alpha value of 0.973.

To collect data, the study selected five provinces, including Hung Yen, Lao Cai, Nghe An, Kien Giang, and Ho Chi Minh City. These provinces were chosen because they are representative of various geographic areas throughout Vietnam, including Northern, Central, and Southern Vietnam, as well as rural, urban, and mountainous areas. Teachers from these provinces, who represent different locality types, ages, and working experiences, participated in the survey. The project information and consent forms were included with the link and sent to the teachers. The study received a total of 30,100 responses, of which 1074 were removed due to missing data. The final dataset analysed included 29,026 responses and was analysed using IBM SPSS Version 26.

## 3 Limitations

This study was conducted through an online questionnaire, which may have posed certain difficulties for teachers in answering the questions, potentially affecting the quality of some responses for teachers with limited access to or proficiency in information technology. Additionally, since this is a self-reported study conducted by teachers, there may be limitations due to self-report bias. However, this limitation does not significantly impact the accuracy and credibility of the data, as the survey was conducted on a large sample, and moreover, the study aimed to explore the challenges that teachers themselves perceive when implementing the new curriculum.

## Ethics Statements

The Ethics Committee of the Vietnam National Institute of Educational Sciences approved and monitored the procedure for conducting this research (ethics approval number: B2022.VKG.05.GRANTED). The procedure for collecting data strictly adhered to the ethical guidelines and regulations of the committee in charge. Teachers were fully informed of the research and asked for their consent before answering the questionnaire. We ensured that the participants' anonymity and confidentiality were maintained throughout the research process.

## CRediT authorship contribution statement

**Hue Thi Thu Dang:** Conceptualization, Methodology, Supervision. **Dien Thi Bui:** Methodology, Writing – original draft, Writing – review & editing. **Quoc Anh Vuong:** Software, Data curation, Validation. **Huong Giang Thi Phan:** Writing – review & editing. **Chi Thi Nguyen:** Writing – review & editing. **Bich Dao Thi Pham:** Methodology, Supervision.

## Declaration of Competing Interest

The authors declare that they have no known competing financial interests or personal relationships that could have appeared to influence the work reported in this paper.

## Data Availability

Dataset from Vietnam: Challenges in Implementing the New National Curriculum from Teachers' Perspective (Original data) (Mendeley Data).
